# Exome sequencing of senescence-accelerated mice (SAM) reveals deleterious mutations in degenerative disease-causing genes

**DOI:** 10.1186/1471-2164-14-248

**Published:** 2013-04-15

**Authors:** Kumpei Tanisawa, Eri Mikami, Noriyuki Fuku, Yoko Honda, Shuji Honda, Ikuro Ohsawa, Masafumi Ito, Shogo Endo, Kunio Ihara, Kinji Ohno, Yuki Kishimoto, Akihito Ishigami, Naoki Maruyama, Motoji Sawabe, Hiroyoshi Iseki, Yasushi Okazaki, Sanae Hasegawa-Ishii, Shiro Takei, Atsuyoshi Shimada, Masanori Hosokawa, Masayuki Mori, Keiichi Higuchi, Toshio Takeda, Mitsuru Higuchi, Masashi Tanaka

**Affiliations:** 1 Department of Genomics for Longevity and Health, Tokyo Metropolitan Institute of Gerontology, 35-2 Sakae-cho, Tokyo, Itabashi, 173-0015, Japan; 2 Graduate School of Sport Sciences, Waseda University, Tokorozawa, 359-1192, Japan; 3 Japan Society for the Promotion of Science, Tokyo, 102-8472, Japan; 4 Department of Biological Process of Aging, Tokyo Metropolitan Institute of Gerontology, Tokyo, 173-0015, Japan; 5 Department of Molecular Gerontology, Tokyo Metropolitan Institute of Gerontology, Tokyo, 173-0015, Japan; 6 Aging Regulation Research Team, Tokyo Metropolitan Institute of Gerontology, Tokyo, 173-0015, Japan; 7 Center for Gene Research, Nagoya University, Nagoya, 464-8602, Japan; 8 Department of Neurogenetics and Bioinformatics, Nagoya University Graduate School of Medicine, Nagoya, 466-8550, Japan; 9 Department of Aging Regulation, Tokyo Metropolitan Institute of Gerontology, Tokyo, 173-0015, Japan; 10 Department of Pathology and Bioresource Center for Geriatric Research, Tokyo Metropolitan Institute of Gerontology, Tokyo, 1730015, Japan; 11 Research Center for Genomic Medicine, Saitama Medical University, Hidaka, 350-1241, Japan; 12 Department of Pathology, Institute for Developmental Research, Aichi Human Service Center, Kasugai, 480-0392, Japan; 13 Department of Aging Biology, Institute on Aging and Adaptation, Shinshu University Graduate School of Medicine, Matsumoto, 390-8621, Japan; 14 The Council for SAM Research, Kyoto, 604-8856, Japan; 15 Faculty of Sport Sciences, Waseda University, Tokorozawa, 359-1192, Japan

**Keywords:** Exome sequencing, Senescence-accelerated mice, Aging

## Abstract

**Background:**

Senescence-accelerated mice (SAM) are a series of mouse strains originally derived from unexpected crosses between AKR/J and unknown mice, from which phenotypically distinct senescence-prone (SAMP) and -resistant (SAMR) inbred strains were subsequently established. Although SAMP strains have been widely used for aging research focusing on their short life spans and various age-related phenotypes, such as immune dysfunction, osteoporosis, and brain atrophy, the responsible gene mutations have not yet been fully elucidated.

**Results:**

To identify mutations specific to SAMP strains, we performed whole exome sequencing of 6 SAMP and 3 SAMR strains. This analysis revealed 32,019 to 38,925 single-nucleotide variants in the coding region of each SAM strain. We detected *Ogg1* p.R304W and *Mbd4* p.D129N deleterious mutations in all 6 of the SAMP strains but not in the SAMR or AKR/J strains. Moreover, we extracted 31 SAMP-specific novel deleterious mutations. In all SAMP strains except SAMP8, we detected a p.R473W missense mutation in the *Ldb3* gene, which has been associated with myofibrillar myopathy. In 3 SAMP strains (SAMP3, SAMP10, and SAMP11), we identified a p.R167C missense mutation in the *Prx* gene, in which mutations causing hereditary motor and sensory neuropathy (Dejerine-Sottas syndrome) have been identified. In SAMP6 we detected a p.S540fs frame-shift mutation in the *Il4ra* gene, a mutation potentially causative of ulcerative colitis and osteoporosis.

**Conclusions:**

Our data indicate that different combinations of mutations in disease-causing genes may be responsible for the various phenotypes of SAMP strains.

## Background

Aging is one of the most complex biological processes that are regulated by both genetic and environmental factors, but its molecular basis remains largely unknown [1]. Senescence-accelerated mice (SAM) are a series of inbred strains developed from the AKR/J strain, consisting of 9 senescence-prone strains (SAMP) and 4 senescence-resistant strains (SAMR) [2,3]. Compared with SAMR strains, which show normal senescence, SAMP strains exhibit accelerated-senescence phenotypes such as a short life span and early onset of various age-related pathological changes [4]. These SAM strains have therefore been used as a model to elucidate the mechanism of aging.

It has remained unknown why SAM strains exhibit different phenotypes, even though they were derived from a common ancestor [2,3]. Genetic analyses by use of biochemical and immunological markers and endogenous murine leukemia virus (MuLV) proviral markers revealed that each SAM strain constitutes a genetically distinct group. Comparisons of the SAM strains with their parental AKR/J strain indicated significant differences in genetic background between them, corroborating the hypothesis of the involvement of other strains, which underscores the probability of accidental outbreeding of the AKR/J strain in the course of the development of SAM [5,6].

Despite intense characterization of SAM strains, the genes responsible for accelerated senescence and pathologic changes in SAMP strains remain unidentified except for mutations in the *Apoa2, Sfrp4, and Fgf1* genes [7-9]. Xia et al. performed genotyping for 581 microsatellite markers in 13 established SAM strains, and identified 4 loci that were different between the SAMP and SAMR strains [10], although the responsible genes remain unknown. Furthermore, genetic analysis of crosses between the SAMP1 and SAMR1 strains also suggested that combinations of multiple gene mutations are responsible for the phenotypes [11].

Recent advances in next-generation sequencing technologies have made it possible to rapidly determine the DNA sequence of the whole genome of individual humans [12,13]. As an alternative approach to whole-genome sequencing, whole-exome sequencing is an efficient strategy with regard to reducing the cost and workload [14,15]. Exome sequencing enables us to obtain information on functionally important coding regions. Although this type of sequencing is useful for identification of the cause of Mendelian disorders [16,17], it is difficult to explore genes responsible for complex traits by using this approach. The difficulty in identification of combined effects of various variants in humans is mainly ascribable to the presence of heterozygosity as well as homozygosity in humans [18]. In contrast, inbred mouse strains such as SAM strains are useful models to analyze the combined effects of genes because we can focus on homozygous variations only.

In the present study, we performed whole exome sequencing of 6 SAMP and 3 SAMR strains to identify the single-nucleotide variations (SNVs) in their entire exomes. We hypothesized that the accelerated-senescence phenotypes and short life span observed in SAMP strains are caused by coding-region mutations that are present specifically in SAMP strains but are absent in the SAMR strains. We obtained a full view of the exome signature of SAM strains and report herein several mutations that potentially cause various pathogenic phenotypes. Our data demonstrate that this innovative approach, whole-exome sequencing, is paving the way to the unraveling of the genetic mechanisms of accelerated senescence and pathogenic phenotypes in mouse models.

## Results

### Whole-exome sequencing revealed exonic profiles of SAM strains

Whole-exome capture and next-generation sequencing were successfully performed on 11 mouse strains, i.e., SAMP1/SkuSlc, SAMP3/SlcIdr, SAMP6/TaSlc, SAMP8/TaSlc, SAMP10/TaSlc, SAMP11/SlcIdr, SAMR1/SlcIdr, SAMR1/TaSlc, SAMR3B/SlcIdr, AKR/J, and C57BL/6J strains, and generated on average 73 million, single-end 50-bp reads per sample (Table 1). The number of aligned reads was about 51 million including 1.7 gigabases of sequence per sample, and the generated sequences achieved a mean read depth of 33.1 ± 11.8×. On average, 91.8 ± 0.4% of the target base pairs were covered by at least one read; and 72.9 ± 6.5% of the target base pairs were covered by at least 10 reads. After removal of low-quality reads, duplicates, and reads mapped out of targeted regions, SNV detection was performed by use of Avadis NGS ver1.3. The whole-exome sequencing identified 85,198 to 112,245 SNVs for SAMP strains, 108,059 to 121,517 for SAMR strains, 100,463 for AKR/J, and 3,484 for C57BL/6J on targeted regions from the reference mouse genome sequence GRCm38 (NCBI37/mm9; Table 2). The number of SNVs in C57BL/6J mice was much lower than that of those in the other strains, because the genome sequence of C57BL/6J was used as the reference for the mouse genome. These 3,484 SNVs in the C57BL/6J strain may be attributed to individual variability rather than to sequencing error. Actually, several studies reported that minor genetic and phenotypic variations could be observed even among individual C57BL/6J mice [19,20]. Although the targeted regions included non-coding regions, we restricted our analysis to exonic SNVs of 32,019 to 35,817 variants for SAMP strains, 36,174 to 38,925 for SAMR strains, 32,816 for AKR/J, and 1,407 for the C57BL/6J strain. Exonic SNVs included 6,507 to 7,843 non-synonymous SNVs for each sample except for C57BL/6J. Moreover, 230 to 491 novel non-synonymous SNVs were detected after comparison with the public database dbSNP128 and genome sequences of 17 inbred strains of laboratory mice [21]. In the same way, novel multiple nucleotide variants (MNV), frame-shift mutations, and nonsense mutations were detected in each strain (Table 2). We calculated the rates of false positive and false negative by validating 32 known and 61 novel SNVs by using the Sanger method (Additional file 1: Tables S1-S3). Although the false-positive rate was 14% for novel SNVs, the entire false-positive rate was 8.0%, indicating that high-quality calls for homozygous SNVs were gained.

**Table 1:** Number of mapped reads and read depth obtained through exome sequencing of 11 mouse strains

**Strains**	**SAMP1/ TaSlc**	**SAMP3/ SlcIdr**	**SAMP6/ TaSlc**	**SAMP8/ TaSlc**	**SAMP10/ TaSlc**	**SAMP11/ SlcIdr**	**SAMR1/ SlcIdr**	**SAMR1/ TaSlc**	**SAMR3B/ SlcIdr**	**AKR/J**	**C57BL/6J**	**Average**
Targeted exons	221,784	221,784	221,784	221,784	221,784	221,784	221,784	221,784	221,784	221,784	221,784	221,784
Target exons with no coverage	7,740	7,132	6,945	7,004	7,238	6,867	6,924	6,332	7,165	7,557	7,384	7,117
Total Target bases	51,555,503	51,555,503	51,555,503	51,555,503	51,555,503	51,555,503	51,555,503	51,555,503	51,555,503	51,555,503	51,555,503	51,555,503
Target bases not covered	4,530,669	4,368,898	4,182,707	4,142,933	4,167,472	3,987,407	4,013,747	3,824,098	4,138,242	4,334,915	4,556,739	4,204,348
Percent of target bases not covered	8.8%	8.5%	8.1%	8.0%	8.1%	7.7%	7.8%	7.4%	8.0%	8.4%	8.8%	8.2%
Total reads	51,745,673	35,778,184	46,935,236	49,808,616	46,543,914	60,395,876	55,759,506	92,605,357	59,459,968	33,681,727	33,076,734	51,435,526
Reads in target regions	37,488,887	30,022,764	38,828,147	39,487,865	35,776,159	44,391,567	43,666,102	76,028,527	42,978,757	23,655,073	24,442,946	39,706,072
Reads off target regions	14,256,786	5,755,420	8,107,089	10,320,751	10,767,755	16,004,309	12,093,404	16,576,830	16,481,211	10,026,654	8,633,788	11,729,454
Percent of reads in target regions	72.4%	83.9%	82.7%	79.3%	76.9%	73.5%	78.3%	82.1%	72.3%	70.2%	73.9%	76.9%
Coverage at 1×	91.2%	91.5%	91.9%	92.0%	91.9%	92.3%	92.2%	92.6%	92.0%	91.6%	91.2%	91.8%
Coverage at 5×	83.0%	81.6%	84.4%	85.2%	84.8%	86.6%	86.5%	89.0%	86.3%	80.5%	79.9%	84.4%
Coverage at 10×	71.4%	67.1%	73.2%	74.6%	73.4%	77.6%	77.4%	84.0%	77.4%	63.1%	62.9%	72.9%
Coverage at 20×	49.6%	41.6%	50.9%	52.9%	50.0%	57.6%	57.2%	72.3%	57.6%	34.6%	35.1%	50.9%
Average depth of coverage within target regions	31.0×	25.3×	32.7×	33.0×	29.6×	37.0×	36.3×	63.7×	35.3×	19.5×	20.2×	33.1×

**Table 2:** Number of SNVs identified through exome sequencing of 11 mouse strains

**Strains**	**SAMP1/ TaSlc**	**SAMP3/ SlcIdr**	**SAMP6/ TaSlc**	**SAMP8/ TaSlc**	**SAMP10/ TaSlc**	**SAMP11/ SlcIdr**	**SAMR1/ SlcIdr**	**SAMR1/ TaSlc**	**SAMR3B/ SlcIdr**	**AKR/J**	**C57BL/6J**
Total SNVs	112245	85198	90734	97039	106341	104290	108059	108880	121527	100463	3484
Exonic SNVs	35791	32019	32659	34048	35752	35817	36174	38432	38925	32816	1407
Homozygous	28916	26142	27914	26142	29282	29310	30253	25891	31338	26880	158
Non-synonymous	7364	6811	7106	7435	7429	7413	7588	6507	7843	6903	63
Novel	310	260	316	273	317	286	317	230	491	245	39
Total MNVs	2251	1610	1757	1870	2093	2007	2207	2210	2245	1834	67
Exonic MNVs	529	433	478	473	528	511	535	571	539	407	22
Homozygous	404	346	375	393	428	412	406	420	417	349	14
Non-synonymous	258	228	243	260	279	273	262	274	294	215	12
Novel	49	33	48	43	50	48	51	63	32	32	8
Total Indels	4439	3189	3653	3932	4446	4286	4749	4287	4943	3988	111
Exonic Indels	470	371	406	413	478	494	505	498	554	411	33
Homozygous	83	64	65	62	69	68	81	77	94	76	3
Frameshift	11	8	8	9	7	8	10	10	14	8	0
Novel	3	2	4	6	2	3	4	3	7	2	0
Total Gain of Stops	242	125	149	155	158	153	177	246	253	132	30
Homozygous	72	56	68	76	61	67	73	57	68	60	1
Novel	10	2	4	6	8	5	10	8	5	1	1

### No novel exonic mutations commonly detected among SAMP strains

Surprisingly, we detected no novel mutations that were present in all of the SAMP strains, but absent in the other strains (Additional file 1: Table S4). When including the SNVs previously reported in other strains, 7 SNVs (*Ogg1* p.R304W, *Tsen2* p.228L, *Mbd4* p.D129N, *D6Wsu116e* p.P556S, *Alox5* p.V95I, *Moxd1* p.R516K, and *Moxd1* p.K583N) were commonly detectable in all of the SAMP strains but absent in SAMR, AKR/J and C57BL/6J strains (Table 3). Having examined the effects of SNVs on protein function, we predicted *Ogg1* p.R304W to be deleterious by both SIFT and PolyPhen-2 programs (SIFT score: 0.00, PolyPhen-2 score: 0.999), and *Mbd4* p.D129N to be deleterious only by the PolyPhen-2 program (SIFT score: 0.12, PolyPhen-2 score: 0.996). We performed functional enrichment analysis to confirm whether common features could be detected among these 6 genes. GO analysis showed that “base-excision repair” (*Ogg1* and *Mbd4)* was most significantly overrepresented (adjusted p-value=0.0003; Table 4). *Ogg1* and *Mbd4* genes were included among the entire top 5 of overrepresented GO categories. Only “response to stress” included *Alox5* in addition to *Ogg1* and *Mbd4* (adjusted p-value=0.0202). We also checked whether these 6 genes had been reported to be associated with the aging process by referring to the GenAge database [22], but no such genes were recorded in the database (data not shown).

**Table 3:** Missense SNVs detected among all of the SAMP strains, but absent in the SAMR and AKR/J strains

**Location**	**Gene symbol**	**Gene name**	**Nucleotide change**	**Amino acid change**	**SIFT score**	**PolyPhen-2 score**	**SNV carriers in 17 inbred strains of laboratory mice**
Chr6:113283636	*Ogg1*	8-oxoguanine DNA-glycosylase 1	c.1125C>T	p.R304W	0	0.999	NOD/ShLtJ
Chr6:115509985	*Tsen2*	tRNA splicing endonuclease 2 homolog (S. cerevisiae)	c.728C>T	p.P228L	0.32	0^*^	129P2/OlaHsd, 129S1/SvImJ, 129S5SvEvBrd, DBA/2J, LP/J, NOD/ShiLtJ, NZO/HlLtJ, WSB/EiJ
Chr6:115799662	*Mbd4*	methyl-CpG binding domain protein 4	c.896C>T	p.D129N	0.12	0.996	129P2/OlaHsd, 129S1/SvImJ, 129S5SvEvBrd, DBA/2J, LP/J, NOD/ShiLtJ, NZO/HlLtJ
Chr6:116186163	*D6Wsu116e (Fam21)*	DNA segment, Chr 6, Wayne State University 116, expressed(WASH complex subunit FAM21)	c.1737C>T	p.P556S	0.73	0.059^*^	129P2/OlaHsd, 129S1/SvImJ, 129S5SvEvBrd, DBA/2J, LP/J, NOD/ShiLtJ, NZO/HlLtJ
Chr6:116360826	*Alox5*	arachidonate 5-lipoxygenase	c.2040C>T	p.V646I	0.07	0	129P2/OlaHsd, 129S1/SvImJ, 129S5SvEvBrd, DBA/2J, LP/J, NOD/ShiLtJ, NZO/HlLtJ
Chr10:24020000	*Moxd1*	monooxygenase, DBH-like 1	c.1634G>A	p.R516K	1	0^*^	NOD/ShiLtJ, WSB/EiJ
Chr10:24021342	*Moxd1*	monooxygenase, DBH-like 1	c.1836A>C	p.K583N	0.39	0.006	NOD/ShiLtJ, WSB/EiJ

^*^Human protein sequence is in conflict with the mouse sequence at the position indicated.

**Table 4:** Top 5 overrepresented GO terms within the 6 genes including missense SNVs detected among all of the SAMP strains, but absent in the SAMR and AKR/J strains

**GO ID**	**GO category**	**Number of reference genes in the category**	**Number of genes in the gene set and also in the category**	**Expected number in genes in the gene set**	**Raw p-value**	**Adjusted p-value**	**Gene symbol**
GO:0006284	base-excision repair	17	2	0.01	1.35×10^-5^	0.0003	*Mbd4*, *Ogg1*
GO:0006281	DNA repair	213	2	0.08	0.0022	0.0202	*Mbd4*, *Ogg1*
GO:0006950	response to stress	1107	3	0.39	0.0042	0.0202	*Alox5*, *Mbd4*, *Ogg1*
GO:0034984	cellular response to DNA damage stimulus	243	2	0.09	0.0028	0.0202	*Mbd4*, *Ogg1*
GO:0006974	response to DNA damage stimulus	274	2	0.1	0.0036	0.0202	*Mbd4*, *Ogg1*

The *Ogg1* p.R304W mutation was previously observed in all of the SAMP strains, but this same mutation was also detected in NZB/N, NFS/N, SJL/J, and NOD/ShiLtJ strains [23]. The *Ogg1* gene encodes the enzyme 8-oxoguanine DNA glycosylase, by which oxidatively modified bases are repaired [24,25]. The methyl-CpG-binding domain protein, encoded by the *Mbd4* gene, is also a DNA repair enzyme that is responsible for removing mismatched thymine or uracil within methylated CpG sites [26]. Similar to *Ogg1* p.R304W, *Mbd4* p.D129N was previously found in normal mice strains including 129P2/OlaHsd, 129S1/SvImJ, 129S5SvEvBrd, DBA/2J, LP/J, NOD/ShiLtJ, and NZO/HlLtJ [21]. It is interesting that all of the SAMP strains as well as the NOD/ShiLtJ strain share these genes that are involved in DNA repair, i.e., *Ogg1* and *Mbd4*. NOD/ShiLtJ is a mouse model of type 1 diabetes, showing a short life span [27,28]. Nevertheless, we should be careful to conclude that the combination of these mutations regulates the accelerated-senescence phenotype of SAMP, because the short life span of NOD/ShiLtJ is generally attributed to diabetes caused by insulitis.

### Unique deleterious mutations identified in each substrain

We hypothesized that different disease phenotypes among SAMP strains are caused by deleterious SNVs that are unique to each strain or a subgroup of strains. We focused on novel non-synonymous SNVs specific to each strain. We extracted SAMP-specific novel non-synonymous SNVs, after excluding mutations in olfactory-receptor and vomeronasal-receptor superfamily genes or in pseudogenes. In addition to nonsense and frameshift mutations, we focused on dysfunctional mutations predicted to be deleterious by SIFT or PolyPhen-2 programs. As the results, we detected 44 deleterious mutations. Subsequently, 31 of these deleterious mutations were validated by Sanger sequencing (Tables 5, 6 and 7, Additional file 1: Tables S5-S9). Among these 31 mutations, only 7 of them were shared by multiple strains (Table 5), whereas the other 24 mutations were detected in only a single strain. Functional enrichment analysis for the genes including these 31 mutations showed that “gap junction channel activity” (*Gja1* and *Gja3*) was the most significantly overrepresented (*p*=0.0043; Additional file 1: Table S10). However, GO category “aging” and its subcategories were not significantly overrepresented. We also confirmed that no genes including these 31 mutations were recorded in the GenAge database (data not shown) [22].

**Table 5:** Novel deleterious mutations detected among multiple SAMP strains, but absent in the SAMR and AKR/J strains

**Location**	**Gene symbol**	**Gene name**	**Nucleotide change (cDNA)**	**Amino acid change**	**Strains**	**SIFT score**	**Polyphen-2 score**
Chr14:35357289	*Ldb3*	LIM domain binding 3	c.1555G>A	p.R473W	SAMP1/SkuSlc,SAMP3/SlcIdr,SAMP6/TaSlc, SAMP10/TaSlc, SAMP11/SlcIdr	0.02	0.968
Chr14:57654538	*Gja3*	gap junction protein, alpha 3	c.1427A>G	p.S405P	SAMP3/SlcIdr,SAMP6/TaSlc, SAMP10/TaSlc, SAMP11/SlcIdr	0.09	0.917^†^
Chr7:28301176	*Prx*	periaxin	c.784C>T	p.R167C	SAMP3/SlcIdr, SAMP10/TaSlc, SAMP11/SlcIdr	0.01	0.998
Chr11:49958661	*Tbc1d9b*	TBC1 domain family, member 9B	c.560T>C	p.S161P	SAMP3/SlcIdr, SAMP10/TaSlc	0	0.867
Chr11:70277672	*Zmynd15*	zinc finger, MYND-type containing 15	c.1859C>T	p.T461M	SAMP3/SlcIdr, SAMP10/TaSlc	0.05	N.A.
Chr18:37455608	*Pcdhb2*	protocadherin beta 2	c.1115G>A	p.G327R	SAMP10/TaSlc, SAMP11/SlcIdr	0	N.A.
Chr18:37495244	*Pcdhb6*	protocadherin beta 6	c.1563G>T	p.E521D	SAMP10/TaSlc, SAMP11/SlcIdr	0	N.A.

^†^Human protein sequence is in conflict with the mouse sequence at the position indicated.

**Table 6:** Novel deleterious mutations specific to SAMP6/TaSlc

**Location**	**Gene symbol**	**Gene name**	**Nucleotide change (cDNA)**	**Amino acid change**	**SIFT score**	**Polyphen-2 score**
Chr2:29947531	*Zdhhc12*	zinc finger, DHHC domain containing 12	c.381G>A	p.R112C	0	0.999
Chr2:31793494	*Lamc3*	laminin gamma 3	c.4202A>G	p.D1380G	0.05	0.399
Chr7:132719753	*Il4ra*	interleukin 4 receptor, alpha	c.1860delC	p.S540fs	N.A.	N.A.
Chr10:79474903	*Abca7*	ATP-binding cassette, sub-family A (ABC1), member 7	c.5477G>A	p.R1826H	0.01	0.108
Chr15:99998371	*Dip2b*	DIP2 disco-interacting protein 2 homolog B	c.1708G>A	p.A544T	0.05	0.17
Chr17:25257897	*Ptx4*	pentraxin 4	c.176A>T	p.R34S	0^*^	0.999
Chr19:4153338	*Coro1b*	coronin, actin-binding protein 1B	c.1253C>T	p.R393W	0	0.998

^*^The score is low confidence because the prediction was based on sequences too closely related.

**Table 7:** Novel deleterious mutations specific to SAMP8/TaSlc

**Location**	**Gene symbol**	**Gene name**	**Nucleotide change (cDNA)**	**Amino acid change**	**SIFT score**	**Polyphen-2 score**
Chr2:60190230	*Ly75*	lymphocyte antigen 75	c.1657G>A	p.R553W	0.02	0.997
Chr5:75023400	*Lnx1*	ligand of numb-protein X 1	c.685T>A	p.N154Y	0	0.318
Chr15:34358663	*Matn2*	matrilin 2	c.2668C>T	p.A806V	0.01	0.717
Chr16:14195230	*Myh11*	myosin, heavy polypeptide 11, smooth muscle	c.5938C>T	p.R1945H	0.17	0.99
Chr16:17506416	*Aifm3*	apoptosis-inducing factor, mitochondrion-associated 3	c.1966G>T	p.K582N	0.01	0.879

We also detected 52 novel deleterious mutations in SAMR strains (Additional file 1: Table S11). These results are not surprising, because it has been reported that SAMR strains exhibit several diseases such as non-thymic lymphoma, histiocytic sarcoma, and ovarian cysts [29], although the SAMR strains have been used as control groups against the SAMP strains. Novel deleterious mutations including *Fbxl13* p.S734N, *Sh3bp5l* p.R217W, *Tnrc6a* p.A278V, and *Zkscan2* p.C232X were detected among all of the SAMP strains in addition to being found in several SAMR strains (Additional file 1: Table S12). These mutations may be associated with susceptibility to diseases in SAMP strains as well as in SAMR ones.

### *Prx* p.R167C mutation in SAMP3, SAMP10, and SAMP11 strains

**Figure 1:**
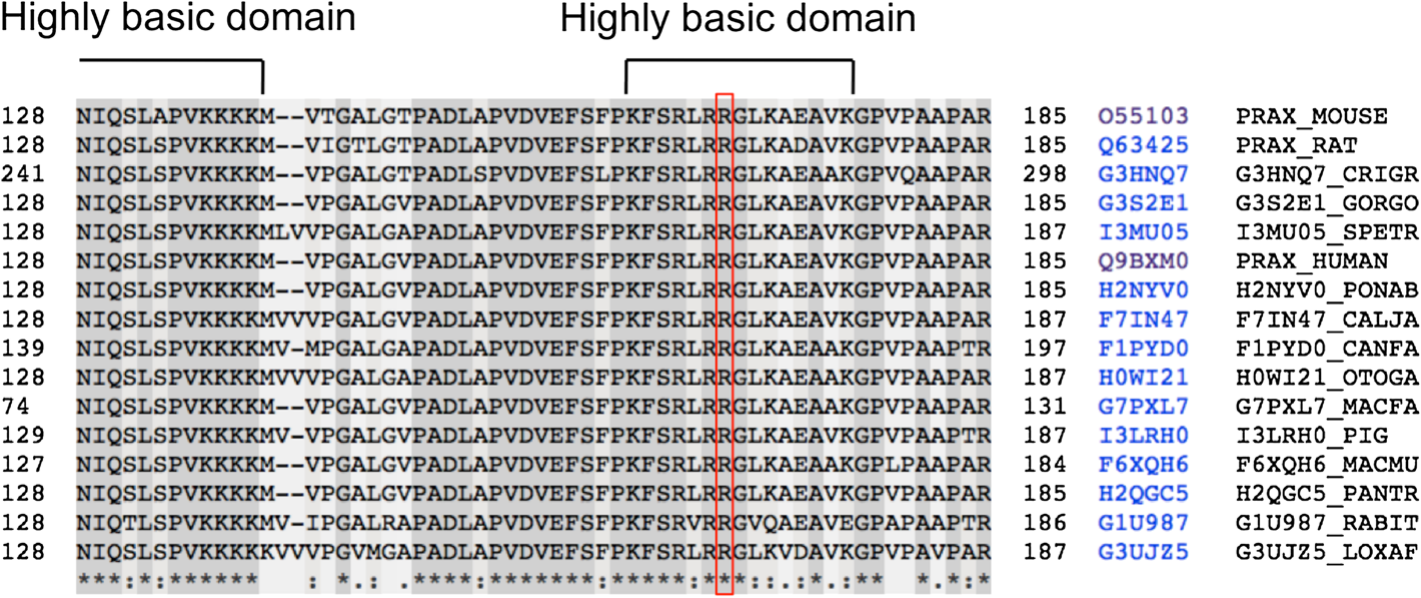
**Multiple sequence alignment of periaxin.** The multiple sequence alignment of periaxin is displayed. The nuclear localization signal of periaxin (amino acids 118–196) comprises 3 highly basic sub-domains. The p.R167C mutation is located in the second sub-domain (amino acid sequence KFSRLRRGLKAEAVK).

Interestingly, we detected deleterious mutations in several genes that had been earlier reported to be associated with severe genetic disorders in both humans and mice. For example, the *Prx* p.R167C mutation (SIFT score: 0.01, PolyPhen-2 score: 0.998) was detected in 3 SAMP strains (SAMP3/SlcIdr, SAMP10/TaSlc, and SAMP11/SlcIder; Table 5). The *Prx* gene encodes periaxin, a protein required for the maintenance of myelin [30]. Because myelin is necessary for the conduction of high-frequency and high-velocity nerve impulses by saltatory conduction, its defects lead to severe neuropathy. In humans, nonsense mutations in periaxin cause an autosomal recessive form of CMT4F (Dejerin-Sottas disease), which is one of the severe hereditary motor and sensory neuropathies [31,32]. It was also reported that periaxin-knockout mice exhibit peripheral demyelination, mechanical allodynia, and thermal hyperalgesia [33]. The *Prx* p.R167C mutation is located within the nuclear localization signal (NLS), which is necessary for this protein to be imported into the nucleus from the cytoplasm (Figure 1) [34]. Localization of periaxin in the nucleus is observed in murine embryonic Schwann cells only for a limited period of time [35]. Because the arginine at position167 is highly conserved among mammalian species, this mutation in NLS might disturb the transfer of periaxin into the nucleus, thereby adversely affecting the normal differentiation of Schwann cells.

### *Ldb3* p.R473W mutation in all of SAMP strains except for SAMP8

In the *Ldb3* gene, encoding LIM domain-binding protein 3, the p.R467W mutation (SIFT score: 0.02, PolyPhen-2 score: 0.968) was detected in all of the SAMP strains except for SAMP8/TaSlc (Table 5). Ldb3 is a component of the sarcomere Z disk protein complex expressed in cardiac and skeletal muscles, and it is connected to calsarcin-1 and α-actinin [36]. Mutations in the *Ldb3* gene are responsible for myofibrillar myopathy and dilated cardiomyopathy in humans [37,38]. In addition, *LDB3* exon 4 is aberrantly spliced in myotonic dystrophy type 1 [39]. Pathological changes in skeletal and cardiac muscles of SAMP strains, however, have not been fully analyzed.

### *Gja3* p.S405P mutation in SAMP3, SAMP6, SAMP10, and SAMP11 strains

We detected the *Gja3* p.S405P mutation (SIFT score: 0.09, PolyPhen-2 score: 0.917) in 4 SAMP strains (SAMP3/SlcIdr, SAMP6/TaSlc, SAMP10/TaSlc and SAMP11/SlcIdr; Table 5). Gap junction protein alpha 3, encoded by *Gja3,* is specifically expressed in the plasma membrane of lens fiber cells to form gap junctions [40]. Gap junctions directly connect the cytoplasm of adjacent cells, and allow various molecules and ions to pass freely between cells, functioning for the maintenance of osmotic and metabolic balance in the avascular lens. A large number of studies have reported the association of mutations of the *GJA3* gene with cataract in humans [41,42].

### *Il4ra* p.S540fs and *Zdhhc12* p.R112C mutations in the SAMP6 strain

**Figure 2:**
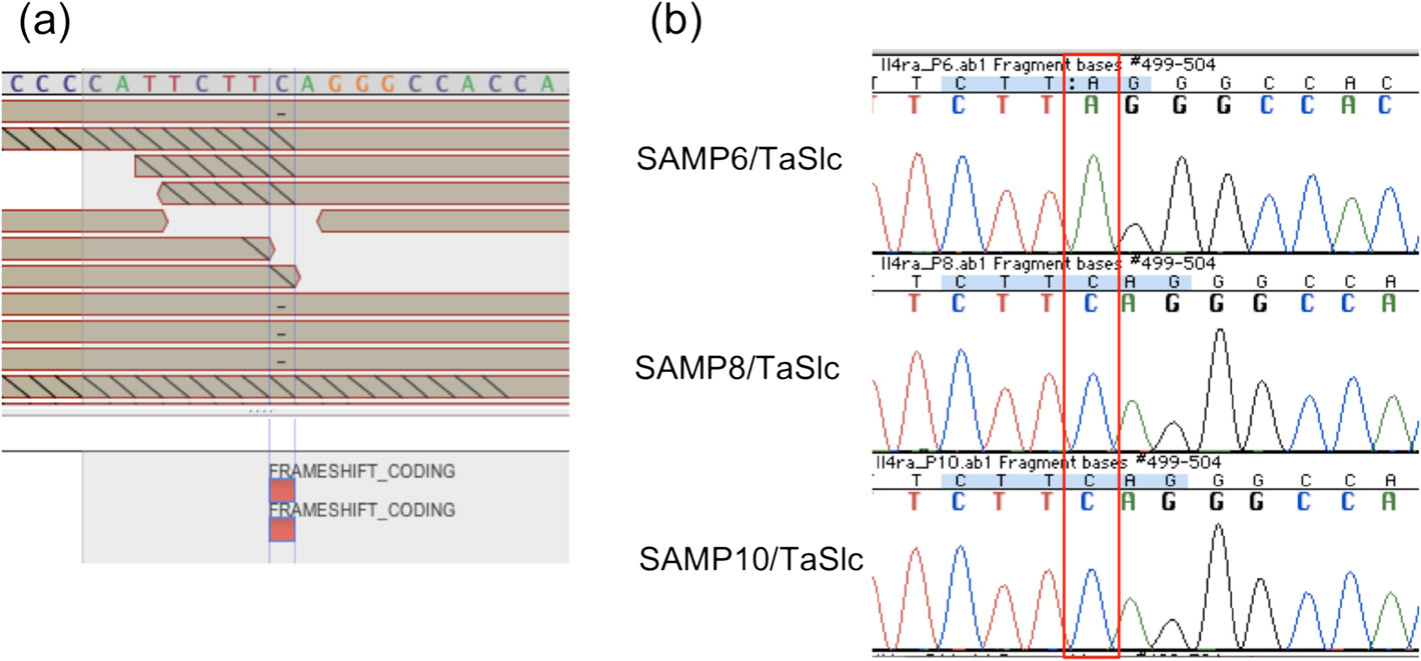
**Visualization of short read mapping on the Avadis NGS genome browser. (a)** The *Il4ra* c.1860delC mutation identified in SAMP6/TaSlc is visualized on the Avadis NGS genome browser. The reference genome sequence GRCm38 (NCBI37/mm9) is shown above, and short DNA reads are aligned to this reference sequence. Hyphens on the reads mean nucleotide deletion at that position. **(b)** Sanger sequencing chromatogram of the *Il4ra* gene is displayed. The *Il4ra* c.1860delC mutation in SAMP6/TaSlc was confirmed by Sanger sequencing.

We next analyzed deleterious SNVs unique to a single SAMP strain, i.e., not shared with other SAMP strains. We detected 7 deleterious mutations specific to the SAMP6/TaSlc strain, which has been used as a mouse model of osteoporosis or ulcerative colitis [43,44]. Among these 7 mutations, we focused on the *Il4ra* p.S540fs frameshift mutation (Figure 2, Table 6). The *Il4ra* p.S540fs substitution immediately generates a stop codon at this position. Osteoporosis is caused by bone resorption in excess of bone formation. The differentiation of osteoclasts is promoted by RANKL, a membrane-bound cytokine, as well as by inflammatory cytokines such as TNF-α, IL-1 and IL-6 [45-48]. These proteins are mainly expressed in type 1 T-helper lymphocytes (Th1 cells). IL-4, a Th2 cytokine suppresses the formation of Th1 cells to keep the proper balance between Th1/Th2 cytokines. A functional loss of the Il4ra protein would lead to activation of Th1 cells. Therefore, both osteoporosis and ulcerative colitis in SAMP6 might be explained by activation of Th1 cytokines, which would be induced by the *Il4ra* p.S540fs frameshift mutation.

The *Zdhhc12* p.R112C mutation (SIFT score: 0.000, PolyPhen-2 score: 0.999) was also detected uniquely in SAMP6 (Table 6). Zinc-finger DHHC domain-containing protein 12, encoded by the *Zdhhc12* gene, has a predicted DHHC cysteine-rich palmitoyl acyltransferase domain [49]. Several gene mutations in the *Zdhhc* family have been implicated in human diseases and abnormal phenotypes of mice. Remarkably, *Zdhhc13-*truncated mutant mice develop alopecia, osteoporosis, and systemic amyloidosis [50]; and the osteoporotic phenotype can be explained by the finding that protein palmitoylation regulates osteoblast differentiation through bone morphogenesis protein (BMP)-induced *Osterix* expression [51]. Thus we speculate that *Zdhhc12* p.R112C mutation might contribute to the osteoporotic phenotype in SAMP6.

### *Aifm3* p.K582N mutation specific to SAMP8

Five detected deleterious mutations were unique to the SAMP8/TaSlc strain, which show deficits in learning and memory, emotional disorder, and abnormal circadian rhythm at early ages (Table 7) [52,53]. It is remarkable that the K582N mutation in the *Aifm3* gene (SIFT score: 0.01, PolyPhen-2 score: 0.879), encoding apoptosis-inducing factor mitochondrion-associated protein 3, was detected in SAMP8/TaSlc. Although the function of Aifm3 has not been fully elucidated, it has been reported that Aifm3 shares 35% homology with Aifm1 and that overexpression of Aifm3 induces apoptosis in HEK 293 cells [54]. Because the lysine at 582 in Aifm3 is highly conserved among mammalian species, this p.K582N mutation therefore may alter the function of Aifm3, contributing to the mitochondrial dysfunction in SAMP8 mice.

## Discussion

### Whole-exome sequencing identified new candidate mutations responsible for age-related phenotypes in SAMP strains

**Figure 3:**
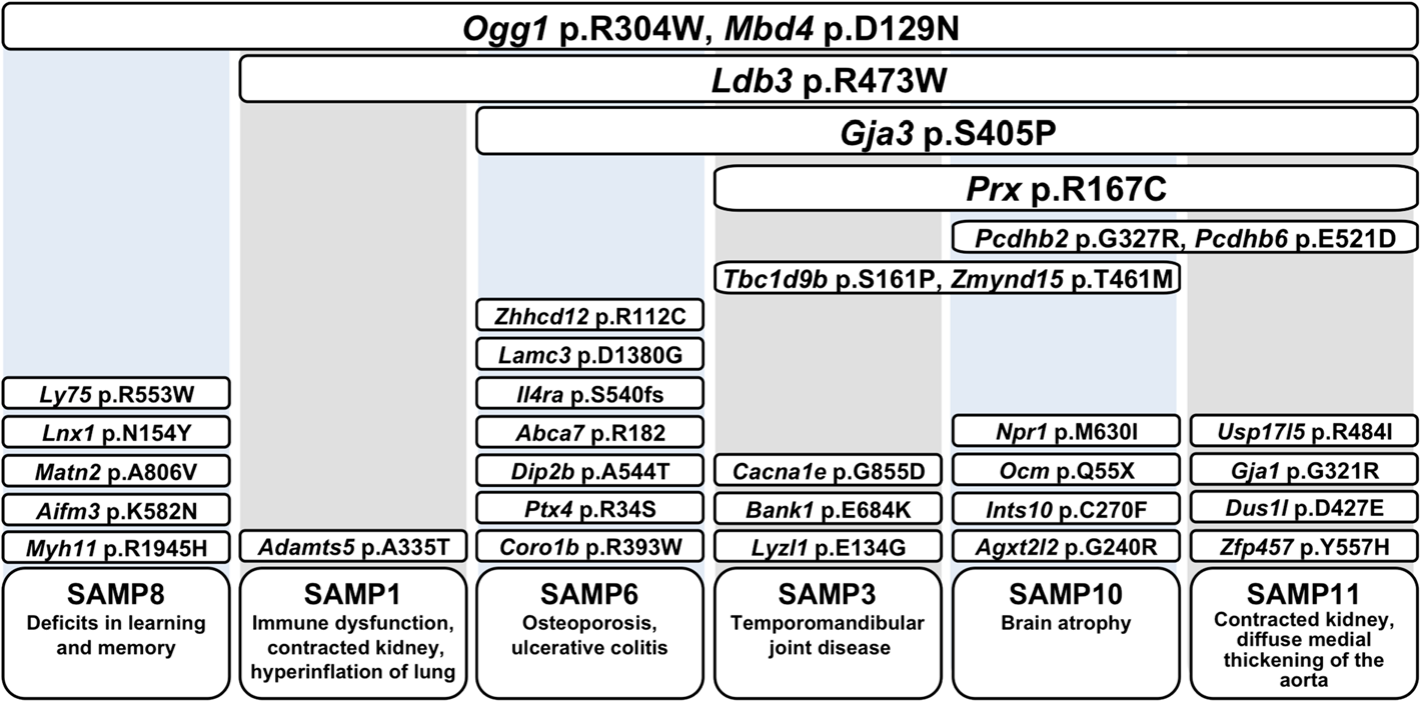
**Summary of candidate mutations regulating various pathogenic phenotypes in SAMP strains.** Described mutations except for *Ogg1* p.R304W and *Mbd4* p.D129N are SAMP-specific novel non-synonymous mutations that were identified in the present study. Although *Ogg1* p.R304W and *Mbd4* p.D129N were detected in other strains, they would be involved in the susceptibility of diseases via defects in DNA repair.

In the present study, we identified the entire spectrum of the SNVs in 6 SAMP and 3 SAMR strains by whole-exome sequencing. We summarized the candidate mutations regulating various pathogenic phenotypes in SAMP strains in Figure 3. Our study has clarified that several disease-causing mutations were common among multiple SAMP strains. Two of these mutations, *Ogg1* p.R304W and *Mbd4* p.D129N, were common among all SAMP strains and would be involved in the susceptibility to diseases via defects in DNA repair. In all SAMP strains except SAMP8/TaSlc, we detected a p.R473W missense mutation in the *Ldb3* gene, which has been associated with myofibrillar myopathy. In 3 SAMP strains (SAMP3/SlcIdr, SAMP10/TaSlc, and SAMP11/SlcIdr), we identified a p.R167C missense mutation in the *Prx* gene, which has been linked to hereditary motor and sensory neuropathy. In 4 SAMP strains (SAMP3/SlcIdr, SAMP6/TaSlc, SAMP10/TaSlc, and SAMP11/SlcIdr), we detected a p.S405P missense mutation in the *Gja3* gene, which is a cause of cataract. As the candidate gene mutations responsible for strain-specific phenotypes, we detected 24 deleterious mutations specific to a single SAMP strain, including the *Il4ra* p.S540fs frameshift mutation in SAMP6/TaSlc, which is used as a model for osteoporosis, and the *Aifm3* p.K582N mutation in SAMP8/TaSlc mice, which display deficits in learning and memory and mitochondrial dysfunction.

We detected *Ogg1* p.R304W and *Mbd4* p.D129N deleterious mutations, which were common to all of the SAMP strains, but absent in the SAMR and AKR/J strains; although these 2 mutations were also detected in other mouse strains. It was already investigated as to whether a defect in Ogg1 protein would affect the life spans in SAMP strains. Mori et al. reported that hybrid mice with the homozygous mutation in *Ogg1* p.R304W exhibited a complete loss of the glycosylase activity as well as a higher level of 8-oxoguanine in their hepatic nuclear DNA [23]. However, the average life span of the SAMP1×B10.BR hybrid was not different among the mice homozygous, heterozygous or nullzygous (B10.BL allele) for the SAMP1 allele. Moreover, NZB/N, NFS/N, SJL/J, and NOD/ShiLtJ also have the *Ogg1* p.R304W mutation. These results suggest that *Ogg1* p.R304W alone is not sufficient to cause accelerated senescence and a short life span. We assume that the combination of *Ogg1* p.R304W and *Mbd4* p.D129N causes accelerated senescence. Both mutations were detected in the NOD/ShiLtJ strain, which is a type 1 diabetes model [27]. Although NOD/ShiLtJ mice may live for only 6 to 8 months due to diabetes under normal food and water conditions [28], we cannot predicate these mutations to be essential for the accelerated-senescence phenotype of SAMP because the cause of death is different between SAMP and NOD/ShiLtJ strains. Nevertheless, mouse strains that possess *Ogg1* p.R304W mutation are known for their pathologic phenotypes: NZB/N for autoimmune hemolytic anemia; SJL/J for reticulum cell sarcomas, in addition to NOD/ShiLtJ for type 1 diabetes [55,56]. Somatic mutations have been implicated in various diseases, and the accumulation of such mutations is one of the most accepted theories to explain aging. The *Ogg1* p.R304W mutation might partly contribute to the phenotypes of these mouse strains as well as to the accelerated-senescence phenotypes of SAMP strains.

In several SAMP strains, missense mutations were detected in the *Prx*, *Ldb3*, and *Gja3* genes, which mutations have been found in various human degenerative diseases. The pathogenesis of myofibrillar myopathy and peripheral neurodegeneration has not been fully analyzed in SAMP strains. Age-related muscle atrophy and a decline in peripheral neuronal function are assumed to be a common phenomenon that probably occurs in the course of the senescence process [57,58]. Nevertheless, genetic susceptibility to degeneration of skeletal muscle and peripheral neurons may be different among SAMP strains. *Prx* p.R167C and *Ldb3* p.R473W mutations possibly contributed to the degenerative phenotypes of 3 of the SAMP strains in the course of the senescence process.

In the present study, the *Gja3* p.S405P mutation was detected in 4 SAMP strains (SAMP3, SAMP6, SAMP10, and SAMP11), among which only the SAMP3 strain is reported to develop cataract [59]. As a lack of reports of cataract in SAMP6, SAMP10, and SAMP11 does not indicate the actual lack of cataract, careful ophthalmologic examinations for cataracts in these 3 other SAMP strains may reveal a pathogenic association. Alternatively, because it is suggested that the pathogenic mechanism underlying the development of cataract in SAMP strains is different from that of murine hereditary cataract, which is generally regulated by single-gene mutations [60], the *Gja3* mutation alone may not be sufficient to cause cataract. The SAMP3 strain may have additional mutations besides the *Gja3* p.S405P mutation that are responsible for cataract.

The *Il4ra* p.S540fs frameshift mutation can explain the osteoporosis observed in the SAMP6 strain from the viewpoint of osteo-immunology. It is known that IL-4 signaling inhibits osteoclast differentiation by suppressing Th1 cytokines such as RANKL, TNF-α, and IL-1. In fact, *Il4* gene knockout mice are sensitive to RANKL-induced bone resorption [61]. A defect in *Il4ra* might thus enhance osteoclast differentiation due to dysregulation of Th1 cytokines. The *Il4ra* p.S540fs frameshift mutation can also explain the ulcerative colitis found in the SAMP6 strain. Although the true cause of ulcerative colitis remains unknown, abnormalities of the immune system are possibly related to its pathogenesis. Particularly, a high level of TNF-α was proposed to play an important role in disease progression [62]. In SAMP6 mice, it is expected that up-regulation of TNF-α expression in the colon would occur due to activation of Th1 cells. Thus, both of these pathogenic phenotypes, osteoporosis and ulcerative colitis, may be ascribable to the defect in *Il4ra* in SAMP6.

We also detected the *Aifm3* p.K582N mutation in the SAMP8/TaSlc mice, which display deficits in learning and memory. High oxidative stress derived from brain mitochondrial dysfunction is thought to be one of the causes of age-related neurodegeneration in SAMP8 animals. Actually, decreased activities of NADH-cytochrome *c* reductase are observed even in 4-week-old SAMP8 mice, suggesting crucial defects in maintenance of the respiratory chain [63]. Aifm3 is likely to be related to mitochondrial maintenance, because it induces apoptosis *in vitro* and has an oxidoreductase domain, as is the case for Aifm1 [54], which plays roles in maintenance of the mitochondrial respiratory chain [64]. However, the actual roles of Aifm3 in apoptosis in the senescence process and the actual substrates of the oxidoreductase remain unknown. Further investigations are necessary to examine whether the *Aifm3* p.K582N contributes to deficits in learning and memory via dysfunction of brain mitochondria.

Overall, it seems that the combinations of different disease-causing mutations specific to each strain cause various degenerative diseases, which combinations are a cause of short life spans of SAMP strains as far as focusing on the mutations of the coding regions is concerned. Actually, it was reported earlier that the life spans of SAMP strains are susceptible to environmental conditions [65]. These observations may be ascribable to the multifactorial nature of the short life span of SAMP unlike other progeroid mice whose life span is regulated by single gene mutation. de Magalhaes JP et al. also reported that the Gompertz mortality curve of the SAMP was not different from that of the SAMR prior to age 1 year despite the difference in age when 50% of mice died, suggesting that the life spans of the SAMP strains may not be related to aging *per se*[66]. Nevertheless, we think that it is premature to conclude that SAMP strains are degenerative disease models rather than accelerated-senescence models because *in vitro* studies have shown that primary-cultured cells from several SAMP strains show accelerated senescence and higher oxidative stress and mitochondrial dysfunction than the SAMR1 strain [67-69].

### Limitation of the present study

Whole-exome sequencing using 50-bp single-end reads on the SOLiD4 platform is able to detect only single or 2-base nucleotide variations and insertion/deletion. Because accelerated senescence and the various pathogenic phenotypes may not be explained completely by the nucleotide substitutions in the coding regions, we cannot ignore the possibility that other types of genetic variations are also involved in common accelerated-senescence phenotypes of SAMP strain. Fairfield et al. succeeded in identifying causative mutations in several ENU-induced mutants by exome sequencing, but failed to do so in several spontaneous disease models [70]. They suggested that mutations responsible for spontaneous disease models might reside in the non-coding regions. Actually, it has been proven that most of the non-coding regions have some biochemical functions [71].

Carter et al. reported a 15-bp insertion mutation in the *Fgf1* gene in SAMP10 [7], suggesting the involvement of a small structural variation in an exon of this gene. A long-read sequencing platform, which can generate over 200-bp fragments, would be required to detect them. It has been suggested that not only small structural variations in exons, but also large genomic structural variations such as copy number variations and gene translocations, contribute to the complex traits of humans [72]. Furthermore, because complementary RNA probes are designed based on reference genome sequences, we were limited to find variants in comparison with the reference sequence. *De novo* assembly by whole genome sequencing or mate-pair library sequencing and comparative genomic hybridization (CGH) array analysis should be performed to detect these sequence variations.

In present study, we focused on only novel deleterious mutations that could be predicted by SIFT and PolyPhen-2. Although these bioinformatics tools are useful to narrow down the candidate mutations, a recent study indicated that SIFT and PolyPhen-2 show 63 and 79% correct prediction rates, respectively [73]. In the future, functional analyses should be conducted to confirm whether the mutations that were predicted to be deleterious in the present study really affect the functions of these genes.

## Conclusions

Our study using whole-exome sequencing provides a list of candidate mutations that are potentially linked with various pathogenic phenotypes. As was shown in Figure 3, 2 deleterious mutations in the DNA-repair genes, i.e., *Ogg1* p.R304W and *Mbd4* p.D129N, were commonly present among SAMP strains, which mutations would be expected to be involved in the genetic vulnerability to age-related diseases. Under such genetic backgrounds, deleterious mutations detected in each substrain may cause various pathogenic phenotypes. We revealed that only 7 SAMP-specific non-synonymous mutations were shared among substrains, although the mechanisms and development of accelerated senescence and short life span have been assumed to be the same among all of SAMP strains. Furthermore, several SAMP strains had deleterious mutations in the genes associated with hereditary diseases (e.g., *Prx* p.R167C, *Ldb3* p.R473W and *Gja3* p.S405P), which mutations have not been previously reported to occur in SAMP strains. These results suggest that comparison of age-related phenotypes among multiple SAMP strains and detailed histopathological reexamination are required. Phenotypic reports of specific SAMP strains have been biased by the researchers’ interests. The current exome sequence data will prompt us to scrutinize yet unnoticed pathological features. In addition to the exome database, construction of the comprehensive genome database of SAMP and SAMR strains will contribute not only to a better understanding of the fundamental aging process occurring in SAM strains but also to elucidation of the mechanisms of age-related diseases in humans as well as to the development of a more effective intervention against them.

## Methods

### DNA extraction

Genomic DNA was extracted from the livers of 11 mouse strains, i.e., SAMP1/SkuSlc, SAMP3/SlcIdr, SAMP6/TaSlc, SAMP8/TaSlc, SAMP10/TaSlc, SAMP11/SlcIdr, SAMR1/SlcIdr, SAMR1/TaSlc, SAMR3B/SlcIdr, AKR/J and C57BL/6J strains. RNase treatment was performed to obtain a high-quality DNA library. All experimental procedures using laboratory animals were approved by the Animal Care and Use Committee of the Tokyo Metropolitan Institute of Gerontology, the Institute for Developmental Research of the Aichi Human Service Center, and by Shinshu University School of Medicine.

### Targeted capture and next-generation sequencing

Target enrichment was performed by use of a SureSelect^XT^ Mouse All Exon kit (Agilent Technologies, Santa Clara, California, US) optimized for the ABI SOLiD system and 3 μg of genomic DNA according to the manufacturer’s protocol. The kit is designed to enrich for 221,784 exons within 24,306 genes covering a total of 49.6 Mb genomic sequences. DNA was sheared by acoustic fragmentation (Covaris, Woburn, Massachusetts, US) and purified with an Agencourt AMPure XP kit (Beckman Coulter, Brea, California, US). The quality of the fragmentation and purification was assessed with an Agilent 2100 Bioanalyzer. The fragment ends were repaired and adaptors were ligated to the fragments (Agilent). The modified DNA library was purified by using the Agencourt AMPure XP kit, and amplified by PCR and captured by hybridization to biotinylated RNA library baits (Agilent). Captured DNA was purified with streptavidin-coated magnetic Dynal beads (Life Technologies, Carlsbad, California, US) and amplified with Barcoding Primer. The prepared exome library was pooled and subjected to emulsion PCR and sequenced on the SOLiD4 (Life Technologies) as single-end 50-bp reads. For each sample, 1 quad of a SOLiD sequencing slide was used.

### Read mapping and variant analysis

Sequence reads were mapped to the reference mouse genome (UCSC mm9, NCBI build 37) by using Bioscope software version 1.3 (Life Technologies), which utilizes an iterative mapping approach. After removal of low-quality and duplicate reads, single nucleotide variants (SNVs) were detected with Avadis NGS software version1.3 (Strand Life Sciences, Bangalore, Karnataka, India). Avadis NGS performs SNV identification via an adapted version of the MAQ algorithm, which calculates the probability that the consensus genotype is incorrect by using a Bayesian statistical model with mapping quality, base quality and ploidy taken into consideration. We established criteria for SNV detection as a read coverage ≥ 2, and other parameters were set as default values. Detected SNVs were annotated for extracting non-synonymous and homozygous SNVs by using the Avadis NGS with UCSC transcript annotation. Moreover, we extracted novel SNVs by comparison with NCBI dbSNP build 128 and SNV data of 17 inbred strains of laboratory mice obtained by whole-genome sequencing. We compared filtered SNVs among all strains to explore the mutations that were commonly present among the SAMP strains but absent in the SAMR strains, AKR/J strain, and C57BL/6J strain. The unique mutations of each strain were also selected.

### Interpretation of novel missense SNVs

To predict whether the candidate SNVs would have deleterious effects or not, we used 2 software programs, i.e., Sorting Intolerant from Tolerant amino acid substitutions (SIFT; J. Craig Venter Institute, San Diego, California, US, http://sift.jcvi.org/) and Polymorphism Phenotyping v2 (PolyPhen-2; Harvard University, Cambridge, Massachusetts, US, http://genetics.bwh.harvard.edu/pph2/). SIFT uses sequence homology to predict amino acid substitutions that will affect protein function, thus contributing to a disease [74]. SIFT predicts substitutions with a score less than 0.05 as being “deleterious“ (Range: 0 to 1). PolyPhen-2 takes into account the physicochemical characteristics of the wild-type and mutated amino acid residue and the consequence of the amino acid change for the structural properties of the protein in addition to evolutional conservation [75]. PolyPhen-2 generates a different scale of reported scores, with the corresponding predictions being “probably damaging” with a score larger than 0.85, “possibly damaging” with a score between 0.85 and 0.15,” and “benign” with a score less than 0.15. Because PolyPhen-2 considers only human protein sequences, the mouse SNVs were investigated in the context of human protein sequences.

### Mutation validation

Validating the candidate SNVs was performed by using the standard Sanger sequencing approach. Primers were designed to surround candidate SNVs by using Primer 3 version 4.0, and custom DNA oligos were ordered (Life Technologies; Operon Biotechnologies, Tokyo, Japan). Primer sequences are shown in Additional file 1: Tables S1-S2. PCR reactions were carried out in 10-μl reaction mixtures containing a 0.5 μM concentration of each primer, 0.2 mM dNTPs, 0.25U Ex Taq DNA Polymerase Hot-Start Version, 1.0 μl 10×Ex Taq Buffer (Takara Bio, Shiga, Japan), and 1 μl of extracted DNA. The amplification conditions were 1 cycle at 96°C for 5 min of denaturation, 40 cycles of 94°C for 30 s, 55-68°C for 45 s of annealing in proportion to the Tm value of each primer, and extension at 72°C for 45 s, followed by a final extension at 72°C for 10 min. PCR products were purified by using a MultiScreen_HTS_ PCR 96-Well Plate (Millipore, Billerica, Massachusetts, US) for sequences. DNA templates were subjected to the sequencing reactions by using a BigDye Terminator version 3.1 Cycle Sequencing Kit (Life Technologies). The sequencing reaction solution contained 4 μl BigDye Terminator v3.1, 0.32 μM M13 forward primer, 1.75 μl 5×Sequence Buffer, and 2.0 μl PCR product in a final volume of 10 μl. PCR conditions were 1 cycle at 94°C of denaturation, 25 cycles of 94°C for 10 s, 50°C for 15 s and 3 min at 60°C, followed by cleaning of the reaction products by ethanol precipitation. The capillary electrophoresis sequencing was performed by using an ABI Prism 3130xl Genetic Analyzer (Life Technologies), and sequence data were analyzed with Sequencher version 4.2.2 (Gene Codes, Ann Arbor, Michigan, US).

### Gene Ontology enrichment analysis

Gene Ontology enrichment analysis (GO analysis) was performed by using WebGestalt (http://bioinfo.vanderbilt.edu/webgestalt/) [76]. The obtained p-values were adjusted by Benjamini-Hochberg multiple testing, and the significant level was established at *p*<0.05.

### Multiple alignment

Multiple sequence alignment was performed by using the Clustal Omega program on the UniProt website (http://www.uniprot.org/) [77].

### Data access

Exome data were deposited in DDBJ Sequence Read Archive (BioProject Accession Number: PRJDB37).

## Competing interests

The authors declare that they have no competing interests.

## Authors’ contributions

KT and MT designed the experiments and drafted the manuscript. KT, EM and NF carried out the exome library preparations. KI carried out the emulsion PCR and the next-generation sequencing. KT carried out the series of bioinformatic analyses including read mapping, variant analysis, interpretation of the SNVs, and multiple alignment. KT carried out the mutation validation by Sanger sequencing. YK, ST, and SHI carried out the sample preparations. KO, HI, and YO supported the bioinformatic analyses. AS, MM, MH, and KH provided the SAM mice. YH, SH, IO, MI, SE, AI, NM, and MS critically reviewed and corrected the manuscript. TT, MH and MT conceived and supervised the entire study. All authors read and approved the final manuscript.

## Supplementary Material

Additional file 1: Table S1 Known SNV list validated by Sanger sequencing, **Table S2.** Novel SNV list validated by Sanger sequencing, **Table S3.** False-positive and false-negative SNV call rate, **Table S4.** Novel SNVs detected among one or more of SAMP strains, but absent in the SAMR and AKR/J strains, **Table S5.** List of false-positive SNVs, **Table S6.** Novel deleterious mutations specific to SAMP1/SkuSlc, We identified only *Adamts5* p.A335T mutation (SIFT score: 0.00, PolyPhen-2 score: 0.810) uniquely in SAMP1/SkuSlc. SAMP1 exhibits senile amyloidosis, impaired immune response, contracted kidney, and lung hyperinflation [78-82]. A disintegrin and metalloproteinase with thrombospondin motifs 5 encoded by *Adamts5* gene functions as an aggrecanase to cleave aggrecan, a major proteoglycan of cartilage [83]. Adamts5 is responsible for aggrecan degradation in a murine model of osteoarthritis [84], and *Adamts5* knockout mice are protected against cartilage degradation through abrogation of joint fibrosis and promoted deposition of cartilage aggrecan [85]. The hyperinflated lungs in SAMP1 result from increased lung compliance, which is related to age-related change in pulmonary elasticity [80]. Although the functions of Adamts5 in tissues except cartilage have not been fully elucidated, the expression of *Adamts5* was also observed in mouse lung. *Adamts5* p.A335T mutation therefore might play roles in hyperinflation of the lungs by affecting the pulmonary elasticity in SAMP1. **Table S7.** Novel deleterious mutations specific to SAMP3/SlcIdr, Three deleterious mutations are specific to SAMP3/SlcIdr, which develops temporomandibular joint disease in an early stage of development [86]. The E684K missense mutation in the *Bank1* gene (SIFT score: 0.13, PolyPhen-2 score: 0.946) encoding B-cell scaffold protein with ankyrin repeats may be associated with temporomandibular joint disease, because polymorphisms in this gene are associated with susceptibility to connective tissue diseases such as rheumatoid arthritis and systemic lupus erythematosus in humans [87,88]. Although the inflammatory status of the temporomandibular condyle in SAMP3 was reported to be similar to that in other SAMP strains [86], we cannot exclude the possibility that the *Bank1* p.E684K mutation contributes to the degenerative changes in the temporomandibular joint in SAMP3. **Table S8.** Novel deleterious mutations specific to SAMP10/TaSlc, We detected 4 deleterious mutations specific to SAMP10/TaSlc. Although both SAMP10 and SAMP8 exhibit memory impairment, SAMP10 is distinct from SAMP8 in that cerebral atrophy occurs specifically in SAMP10 [89,90]. The *Npr1* p.M630I mutation (SIFT score; 0.03, PolyPhen-2 score; 0.939) was detected uniquely in the SAMP10. *Npr1* encodes natriuretic peptide receptor 1 (NPR-A), which is a membrane-bound guanylate cyclase that serves as the receptor for both atrial natriuretic peptide (ANP) and brain natriuretic peptides (BNP) [91]. The main role of ANP/BNP signaling through NPR-A is decreasing systemic vascular resistance and blood pressure via increasing natriuresis [92,93]. The functions of natriuretic peptides in the nervous system also have been examined. NPR-A is mainly localized in glial cells but not in neuronal cells in several regions of the brain [94,95]. Although the cytokine-mediated neuroprotective glial responses are impaired in SAMP10 [96], further examination will be required to elucidate whether the *Npr1* p.M630I contributes to the degenerative brain disorder in SAMP10. **Table S9.** Novel deleterious mutations specific to SAMP11/SlcIdr, Four deleterious mutations are specific to SAMP11/SlcIdr, which exhibits senile amyloidosis and contracted kidney, as well as diffuse medial thickening of the aorta [3,97]. We identified the G321R mutation in the *Gja1* gene (SIFT score: 0.20, PolyPhen-2 score: 1.000) encoding gap junction protein, alpha 1, which is a component of intercellular channels connecting adjacent cells [98]. Gja1 is the major protein of gap junctions in the heart [99]. The mutations in *GJA1* were proved to be the cause of several heart diseases [100,101]. Gja1 protein is also expressed in vascular smooth muscle and is necessary for vascular formation and maintaining vascular function [102,103]. Although the effect of a defect of Gja1 protein on vascular morphology is not consistent among studies, Liao et al. reported that the carotid arteries in smooth muscle cell-specific *Gja1* gene knockout mice thickens after injury more extensively than those of wild-type mice [104], suggesting that disruption of normal gap junctional communication contributes to abnormal vascular phenotypes including diffuse thickening of the aorta in SAMP11. **Table S10.** Top 5 overrepresented GO terms within the 31 genes including novel deleterious mutations detected among one or more of SAMP strains, but absent in the SAMR and AKR/J strains, **Table S11.** Novel deleterious mutations detected among one or more of SAMP and SAMR strains, but absent in AKR/J strain, **Table S12.** Novel deleterious mutations detected among all of the SAMPstrains and several SAMR strains, but absent in the AKR/J strain. Click here for file

## References

[B1] Johnson FB Sinclair DA Guarente L Molecular biology of aging Cell 1999 96 2 291 302 10.1016/S0092-8674(00)80567-X 9988222

[B2] Higuchi K Genetic characterization of senescence-accelerated mouse (SAM) Exp Gerontol 1997 32 1–2 129 138 9088910 10.1016/s0531-5565(96)00060-5

[B3] Takeda T Hosokawa M Higuchi K Senescence-accelerated mouse (SAM): a novel murine model of senescence Exp Gerontol 1997 32 1–2 105 109 9088907 10.1016/s0531-5565(96)00036-8

[B4] Takeda T Matsushita T Kurozumi M Takemura K Higuchi K Hosokawa M Pathobiology of the senescence-accelerated mouse (SAM) Exp Gerontol 1997 32 1–2 117 127 9088909 10.1016/s0531-5565(96)00068-x

[B5] Kitado H Higuchi K Takeda T Molecular genetic characterization of the senescence-accelerated mouse (SAM) strains J Gerontol 1994 49 6 B247 B254 10.1093/geronj/49.6.B247 7963272

[B6] Takeda T Hosokawa M Higuchi K Takeda T Senescence-accelerated mouse (SAM). A novel murine model of aging The SAM model of senescence 1994 Amsterdam: Elsevier B. V 15

[B7] Carter TA Greenhall JA Yoshida S Fuchs S Helton R Swaroop A Lockhart DJ Barlow C Mechanisms of aging in senescence-accelerated mice Genome Biol 2005 6 6 R48 10.1186/gb-2005-6-6-r48 15960800 PMC1175968

[B8] Higuchi K Kitagawa K Naiki H Hanada K Hosokawa M Takeda T Polymorphism of apolipoprotein A-II (apoA-II) among inbred strains of mice. Relationship between the molecular type of apoA-II and mouse senile amyloidosis Biochem J 1991 279 Pt 2 427 433 1683229 10.1042/bj2790427 PMC1151622

[B9] Nakanishi R Shimizu M Mori M Akiyama H Okudaira S Otsuki B Hashimoto M Higuchi K Hosokawa M Tsuboyama T Nakamura T Secreted frizzled-related protein 4 is a negative regulator of peak BMD in SAMP6 mice J Bone Miner Res 2006 21 11 1713 1721 10.1359/jbmr.060719 17002585

[B10] Xia C Higuchi K Shimizu M Matsushita T Kogishi K Wang J Chiba T Festing MF Hosokawa M Genetic typing of the senescence-accelerated mouse (SAM) strains with microsatellite markers Mamm Genome 1999 10 3 235 238 10.1007/s003359900979 10051317

[B11] Naiki H Higuchi K Shimada A Takeda T Nakakuki K Genetic analysis of murine senile amyloidosis Lab Invest 1993 68 3 332 337 8095565

[B12] Wang J Wang W Li R Li Y Tian G Goodman L Fan W Zhang J Li J Guo Y Feng B Li H Lu Y Fang X Liang H Du Z Li D Zhao Y Hu Y Yang Z Zheng H Hellmann I Inouye M Pool J Yi X Zhao J Duan J Zhou Y Qin J Ma L The diploid genome sequence of an Asian individual Nature 2008 456 7218 60 65 10.1038/nature07484 18987735 PMC2716080

[B13] Wheeler DA Srinivasan M Egholm M Shen Y Chen L McGuire A He W Chen YJ Makhijani V Roth GT Gomes X Tartaro K Niazi F Turcotte CL Irzyk GP Lupski JR Chinault C Song XZ Liu Y Yuan Y Nazareth L Qin X Muzny DM Margulies M Weinstock GM Gibbs RA Rothberg JM The complete genome of an individual by massively parallel DNA sequencing Nature 2008 452 7189 872 876 10.1038/nature06884 18421352

[B14] Li Y Vinckenbosch N Tian G Huerta-Sanchez E Jiang T Jiang H Albrechtsen A Andersen G Cao H Korneliussen T Grarup N Guo Y Hellman I Jin X Li Q Liu J Liu X Sparso T Tang M Wu H Wu R Yu C Zheng H Astrup A Bolund L Holmkvist J Jorgensen T Kristiansen K Schmitz O Schwartz TW Resequencing of 200 human exomes identifies an excess of low-frequency non-synonymous coding variants Nat Genet 2010 42 11 969 972 10.1038/ng.680 20890277

[B15] Ng SB Turner EH Robertson PD Flygare SD Bigham AW Lee C Shaffer T Wong M Bhattacharjee A Eichler EE Bamshad M Nickerson DA Shendure J Targeted capture and massively parallel sequencing of 12 human exomes Nature 2009 461 7261 272 276 10.1038/nature08250 19684571 PMC2844771

[B16] Bilguvar K Ozturk AK Louvi A Kwan KY Choi M Tatli B Yalnizoglu D Tuysuz B Caglayan AO Gokben S Kaymakcalan H Barak T Bakircioglu M Yasuno K Ho W Sanders S Zhu Y Yilmaz S Dincer A Johnson MH Bronen RA Kocer N Per H Mane S Pamir MN Yalcinkaya C Kumandas S Topcu M Ozmen M Sestan N Whole-exome sequencing identifies recessive WDR62 mutations in severe brain malformations Nature 2010 467 7312 207 210 10.1038/nature09327 20729831 PMC3129007

[B17] Ng SB Buckingham KJ Lee C Bigham AW Tabor HK Dent KM Huff CD Shannon PT Jabs EW Nickerson DA Shendure J Bamshad MJ Exome sequencing identifies the cause of a mendelian disorder Nat Genet 2010 42 1 30 35 10.1038/ng.499 19915526 PMC2847889

[B18] Feng BJ Tavtigian SV Southey MC Goldgar DE Design considerations for massively parallel sequencing studies of complex human disease PLoS One 2011 6 8 e23221 10.1371/journal.pone.0023221 21850262 PMC3151293

[B19] Jakovcevski M Schachner M Morellini F Individual variability in the stress response of C57BL/6J male mice correlates with trait anxiety Genes Brain Behav 2008 7 2 235 243 10.1111/j.1601-183X.2007.00345.x 17680803

[B20] Watkins-Chow DE Pavan WJ Genomic copy number and expression variation within the C57BL/6J inbred mouse strain Genome Res 2008 18 1 60 66 18032724 10.1101/gr.6927808 PMC2134784

[B21] Keane TM Goodstadt L Danecek P White MA Wong K Yalcin B Heger A Agam A Slater G Goodson M Furlotte NA Eskin E Nellaker C Whitley H Cleak J Janowitz D Hernandez-Pliego P Edwards A Belgard TG Oliver PL McIntyre RE Bhomra A Nicod J Gan X Yuan W van der Weyden L Steward CA Bala S Stalker J Mott R Mouse genomic variation and its effect on phenotypes and gene regulation Nature 2011 477 7364 289 294 10.1038/nature10413 21921910 PMC3276836

[B22] de Magalhaes JP Toussaint O GenAge: a genomic and proteomic network map of human ageing FEBS Lett 2004 571 1–3 243 247 15280050 10.1016/j.febslet.2004.07.006

[B23] Mori M Toyokuni S Kondo S Kasai H Naiki H Toichi E Hosokawa M Higuchi K Spontaneous loss-of-function mutations of the 8-oxoguanine DNA glycosylase gene in mice and exploration of the possible implication of the gene in senescence Free Radic Biol Med 2001 30 10 1130 1136 10.1016/S0891-5849(01)00511-1 11369503

[B24] Nash HM Bruner SD Scharer OD Kawate T Addona TA Spooner E Lane WS Verdine GL Cloning of a yeast 8-oxoguanine DNA glycosylase reveals the existence of a base-excision DNA-repair protein superfamily Curr Biol 1996 6 8 968 980 10.1016/S0960-9822(02)00641-3 8805338

[B25] Thomas D Scot AD Barbey R Padula M Boiteux S Inactivation of OGG1 increases the incidence of G . C-->T . A transversions in Saccharomyces cerevisiae: evidence for endogenous oxidative damage to DNA in eukaryotic cells Mol Gen Genet 1997 254 2 171 178 10.1007/s004380050405 9108279

[B26] Hendrich B Hardeland U Ng HH Jiricny J Bird A The thymine glycosylase MBD4 can bind to the product of deamination at methylated CpG sites Nature 1999 401 6750 301 304 10.1038/45843 10499592

[B27] Makino S Kunimoto K Muraoka Y Mizushima Y Katagiri K Tochino Y Breeding of a non-obese, diabetic strain of mice Jikken Dobutsu 1980 29 1 1 13 6995140 10.1538/expanim1978.29.1_1

[B28] Threadgill DW Miller DR Churchill GA de Villena FP The collaborative cross: a recombinant inbred mouse population for the systems genetic era ILAR J 2011 52 1 24 31 10.1093/ilar.52.1.24 21411855

[B29] Takeda T Senescence-accelerated mouse (SAM): a biogerontological resource in aging research Neurobiol Aging 1999 20 2 105 110 10.1016/S0197-4580(99)00008-1 10537019

[B30] Gillespie CS Sherman DL Blair GE Brophy PJ Periaxin, a novel protein of myelinating Schwann cells with a possible role in axonal ensheathment Neuron 1994 12 3 497 508 10.1016/0896-6273(94)90208-9 8155317

[B31] Guilbot A Williams A Ravise N Verny C Brice A Sherman DL Brophy PJ LeGuern E Delague V Bareil C Megarbane A Claustres M A mutation in periaxin is responsible for CMT4F, an autosomal recessive form of Charcot-Marie-Tooth disease Hum Mol Genet 2001 10 4 415 421 10.1093/hmg/10.4.415 11157804

[B32] Otagiri T Sugai K Kijima K Arai H Sawaishi Y Shimohata M Hayasaka K Periaxin mutation in Japanese patients with Charcot-Marie-Tooth disease J Hum Genet 2006 51 7 625 628 10.1007/s10038-006-0408-3 16770524

[B33] Gillespie CS Sherman DL Fleetwood-Walker SM Cottrell DF Tait S Garry EM Wallace VC Ure J Griffiths IR Smith A Brophy PJ Peripheral demyelination and neuropathic pain behavior in periaxin-deficient mice Neuron 2000 26 2 523 531 10.1016/S0896-6273(00)81184-8 10839370

[B34] Dingwall C Sharnick SV Laskey RA A polypeptide domain that specifies migration of nucleoplasmin into the nucleus Cell 1982 30 2 449 458 10.1016/0092-8674(82)90242-2 6814762

[B35] Sherman DL Brophy PJ A tripartite nuclear localization signal in the PDZ-domain protein L-periaxin J Biol Chem 2000 275 7 4537 4540 10.1074/jbc.275.7.4537 10671475

[B36] Zhou Q Ruiz-Lozano P Martone ME Chen J Cypher, a striated muscle-restricted PDZ and LIM domain-containing protein, binds to alpha-actinin-2 and protein kinase C J Biol Chem 1999 274 28 19807 19813 10.1074/jbc.274.28.19807 10391924

[B37] Selcen D Engel AG Mutations in ZASP define a novel form of muscular dystrophy in humans Ann Neurol 2005 57 2 269 276 10.1002/ana.20376 15668942

[B38] Vatta M Mohapatra B Jimenez S Sanchez X Faulkner G Perles Z Sinagra G Lin JH Vu TM Zhou Q Bowles KR Di Lenarda A Schimmenti L Fox M Chrisco MA Murphy RT McKenna W Elliott P Bowles NE Chen J Valle G Towbin JA Mutations in Cypher/ZASP in patients with dilated cardiomyopathy and left ventricular non-compaction J Am Coll Cardiol 2003 42 11 2014 2027 10.1016/j.jacc.2003.10.021 14662268

[B39] Yamashita Y Matsuura T Shinmi J Amakusa Y Masuda A Ito M Kinoshita M Furuya H Abe K Ibi T Sahashi K Ohno K Four parameters increase the sensitivity and specificity of the exon array analysis and disclose 25 novel aberrantly spliced exons in myotonic dystrophy J Hum Genet 2012 57 6 368 374 10.1038/jhg.2012.37 22513715

[B40] Paul DL Ebihara L Takemoto LJ Swenson KI Goodenough DA Connexin46, a novel lens gap junction protein, induces voltage-gated currents in nonjunctional plasma membrane of Xenopus oocytes J Cell Biol 1991 115 4 1077 1089 10.1083/jcb.115.4.1077 1659572 PMC2289939

[B41] Bennett TM Mackay DS Knopf HL Shiels A A novel missense mutation in the gene for gap-junction protein alpha3 (GJA3) associated with autosomal dominant "nuclear punctate" cataracts linked to chromosome 13q Mol Vis 2004 10 376 382 15208569

[B42] Bennett TM Shiels A A recurrent missense mutation in GJA3 associated with autosomal dominant cataract linked to chromosome 13q Mol Vis 2011 17 2255 2262 21897748 PMC3164684

[B43] Matsushita M Tsuboyama T Kasai R Okumura H Yamamuro T Higuchi K Kohno A Yonezu T Utani A Umezawa M Takeda T Age-related changes in bone mass in the senescence-accelerated mouse (SAM). SAM-R/3 and SAM-P/6 as new murine models for senile osteoporosis Am J Pathol 1986 125 2 276 283 3789087 PMC1888250

[B44] Tanaka S Shiokawa K Miyaishi O Nomura Y Effects of housing and nutritions condition on the reproductions of SAMR1, SAMP6 and SAMP8 at NILS aging farm The Senescence-Accelerated Mouse (SAM): An Animal Model of Senescence 2004 Amsterdam: Elsevier B. V 167 173

[B45] Ishimi Y Miyaura C Jin CH Akatsu T Abe E Nakamura Y Yamaguchi A Yoshiki S Matsuda T Hirano T IL-6 is produced by osteoblasts and induces bone resorption J Immunol 1990 145 10 3297 3303 2121824

[B46] Takahashi N Udagawa N Suda T A new member of tumor necrosis factor ligand family, ODF/OPGL/TRANCE/RANKL, regulates osteoclast differentiation and function Biochem Biophys Res Commun 1999 256 3 449 455 10.1006/bbrc.1999.0252 10080918

[B47] Thomson BM Mundy GR Chambers TJ Tumor necrosis factors alpha and beta induce osteoblastic cells to stimulate osteoclastic bone resorption J Immunol 1987 138 3 775 779 3805716

[B48] Thomson BM Saklatvala J Chambers TJ Osteoblasts mediate interleukin 1 stimulation of bone resorption by rat osteoclasts J Exp Med 1986 164 1 104 112 10.1084/jem.164.1.104 3487611 PMC2188199

[B49] Korycka J Lach A Heger E Boguslawska DM Wolny M Toporkiewicz M Augoff K Korzeniewski J Sikorski AF Human DHHC proteins: a spotlight on the hidden player of palmitoylation Eur J Cell Biol 2011 91 2 107 117 22178113 10.1016/j.ejcb.2011.09.013

[B50] Saleem AN Chen YH Baek HJ Hsiao YW Huang HW Kao HJ Liu KM Shen LF Song IW Tu CP Wu JY Kikuchi T Justice MJ Yen JJ Chen YT Mice with alopecia, osteoporosis, and systemic amyloidosis due to mutation in Zdhhc13, a gene coding for palmitoyl acyltransferase PLoS Genet 2010 6 6 e1000985 10.1371/journal.pgen.1000985 20548961 PMC2883605

[B51] Leong WF Zhou T Lim GL Li B Protein palmitoylation regulates osteoblast differentiation through BMP-induced osterix expression PLoS One 2009 4 1 e4135 10.1371/journal.pone.0004135 19125191 PMC2607547

[B52] Miyamoto M Kiyota Y Nishiyama M Nagaoka A Senescence-accelerated mouse (SAM): age-related reduced anxiety-like behavior in the SAM-P/8 strain Physiol Behav 1992 51 5 979 985 10.1016/0031-9384(92)90081-C 1615059

[B53] Miyamoto M Kiyota Y Yamazaki N Nagaoka A Matsuo T Nagawa Y Takeda T Age-related changes in learning and memory in the senescence-accelerated mouse (SAM) Physiol Behav 1986 38 3 399 406 10.1016/0031-9384(86)90112-5 3786521

[B54] Xie Q Lin T Zhang Y Zheng J Bonanno JA Molecular cloning and characterization of a human AIF-like gene with ability to induce apoptosis J Biol Chem 2005 280 20 19673 19681 10.1074/jbc.M409517200 15764604

[B55] Carswell EA Wanebo HJ Old LJ Boyse EA Immunogenic properties of reticulum cell sarcomas of SJL/J mice J Natl Cancer Inst 1970 44 6 1281 1288 11515449

[B56] Holmes MC Burnet FM The Natural History of Autoimmune Disease in Nzb Mice. A Comparison with the Pattern of Human Autoimmune Manifestations Ann Intern Med 1963 59 265 276 10.7326/0003-4819-59-3-265 14065944

[B57] Frontera WR Hughes VA Fielding RA Fiatarone MA Evans WJ Roubenoff R Aging of skeletal muscle: a 12-yr longitudinal study J Appl Physiol 2000 88 4 1321 1326 10749826 10.1152/jappl.2000.88.4.1321

[B58] Verdu E Ceballos D Vilches JJ Navarro X Influence of aging on peripheral nerve function and regeneration J Peripher Nerv Syst 2000 5 4 191 208 10.1046/j.1529-8027.2000.00026.x 11151980

[B59] Hosokawa M Takeshita S Higuchi K Shimizu K Irino M Toda K Honma A Matsumura A Yasuhira K Takeda T Cataract and other ophthalmic lesions in senescence accelerated mouse (SAM). Morphology and incidence of senescence associated ophthalmic changes in mice Exp Eye Res 1984 38 2 105 114 10.1016/0014-4835(84)90095-2 6714329

[B60] Nishimoto H Uga S Miyata M Ishikawa S Yamashita K Morphological study of the cataractous lens of the senescence accelerated mouse Graefes Arch Clin Exp Ophthalmol 1993 231 12 722 728 10.1007/BF00919288 8299981

[B61] Mangashetti LS Khapli SM Wani MR IL-4 inhibits bone-resorbing activity of mature osteoclasts by affecting NF-kappa B and Ca2+ signaling J Immunol 2005 175 2 917 925 16002690 10.4049/jimmunol.175.2.917

[B62] Sands BE Kaplan GG The role of TNFalpha in ulcerative colitis J Clin Pharmacol 2007 47 8 930 941 10.1177/0091270007301623 17567930

[B63] Fujibayashi Y Yamamoto S Waki A Konishi J Yonekura Y Increased mitochondrial DNA deletion in the brain of SAMP8, a mouse model for spontaneous oxidative stress brain Neurosci Lett 1998 254 2 109 112 10.1016/S0304-3940(98)00667-3 9779932

[B64] Cheung EC Joza N Steenaart NA McClellan KA Neuspiel M McNamara S MacLaurin JG Rippstein P Park DS Shore GC McBride HM Penninger JM Slack RS Dissociating the dual roles of apoptosis-inducing factor in maintaining mitochondrial structure and apoptosis EMBO J 2006 25 17 4061 4073 10.1038/sj.emboj.7601276 16917506 PMC1560366

[B65] Takeda T Nomura Y Effects of environment on life span and pathobiological phenotypes in senescence-accelerated mice The Senescence-Accelerated Mouse (SAM): An Animal Model of Senescence 2004 Amsterdam: Elsevier B. V 3 12

[B66] de Magalhaes JP Cabral JA Magalhaes D The influence of genes on the aging process of mice: a statistical assessment of the genetics of aging Genetics 2005 169 1 265 274 15466429 10.1534/genetics.104.032292 PMC1448866

[B67] Chiba Y Yamashita Y Ueno M Fujisawa H Hirayoshi K Hohmura K Tomimoto H Akiguchi I Satoh M Shimada A Hosokawa M Cultured murine dermal fibroblast-like cells from senescence-accelerated mice as in vitro models for higher oxidative stress due to mitochondrial alterations J Gerontol A Biol Sci Med Sci 2005 60 9 1087 1098 10.1093/gerona/60.9.1087 16183946

[B68] Hosokawa M Ashida Y Nishikawa T Takeda T Accelerated aging of dermal fibroblast-like cells from senescence-accelerated mouse (SAM). 1. Acceleration of population aging in vitro Mech Ageing Dev 1994 74 1–2 65 77 7934209 10.1016/0047-6374(94)90099-x

[B69] Lecka-Czernik B Moerman EJ Shmookler Reis RJ Lipschitz DA Cellular and molecular biomarkers indicate precocious in vitro senescence in fibroblasts from SAMP6 mice. Evidence supporting a murine model of premature senescence and osteopenia J Gerontol A Biol Sci Med Sci 1997 52 6 B331 9402934 10.1093/gerona/52a.6.b331

[B70] Fairfield H Gilbert GJ Barter M Corrigan RR Curtain M Ding Y D'Ascenzo M Gerhardt DJ He C Huang W Richmond T Rowe L Probst FJ Bergstrom DE Murray SA Bult C Richardson J Kile BT Gut I Hager J Sigurdsson S Mauceli E Di Palma F Lindblad-Toh K Cunningham ML Cox TC Justice MJ Spector MS Lowe SW Albert T Mutation discovery in mice by whole exome sequencing Genome Biol 2011 12 9 R86 10.1186/gb-2011-12-9-r86 21917142 PMC3308049

[B71] Dunham I Kundaje A Aldred SF Collins PJ Davis CA Doyle F Epstein CB Frietze S Harrow J Kaul R Khatun J Lajoie BR Landt SG Lee BK Pauli F Rosenbloom KR Sabo P Safi A Sanyal A Shoresh N Simon JM Song L Trinklein ND Altshuler RC Birney E Brown JB Cheng C Djebali S Dong X Ernst J An integrated encyclopedia of DNA elements in the human genome Nature 2012 489 7414 57 74 10.1038/nature11247 22955616 PMC3439153

[B72] Sebat J Lakshmi B Troge J Alexander J Young J Lundin P Maner S Massa H Walker M Chi M Navin N Lucito R Healy J Hicks J Ye K Reiner A Gilliam TC Trask B Patterson N Zetterberg A Wigler M Large-scale copy number polymorphism in the human genome Science 2004 305 5683 525 528 10.1126/science.1098918 15273396

[B73] Gray VE Kukurba KR Kumar S Performance of computational tools in evaluating the functional impact of laboratory-induced amino acid mutations Bioinformatics 2012 28 16 2093 2096 10.1093/bioinformatics/bts336 22685075 PMC3413386

[B74] Ng PC Henikoff S SIFT: Predicting amino acid changes that affect protein function Nucleic Acids Res 2003 31 13 3812 3814 10.1093/nar/gkg509 12824425 PMC168916

[B75] Ramensky V Bork P Sunyaev S Human non-synonymous SNPs: server and survey Nucleic Acids Res 2002 30 17 3894 3900 10.1093/nar/gkf493 12202775 PMC137415

[B76] Zhang B Kirov S Snoddy J WebGestalt: an integrated system for exploring gene sets in various biological contexts Nucleic Acids Res 2005 33 Web Server issue W741 W748 15980575 10.1093/nar/gki475 PMC1160236

[B77] Sievers F Wilm A Dineen D Gibson TJ Karplus K Li W Lopez R McWilliam H Remmert M Soding J Thompson JD Higgins DG Fast, scalable generation of high-quality protein multiple sequence alignments using Clustal Omega Mol Syst Biol 2011 7 539 21988835 10.1038/msb.2011.75 PMC3261699

[B78] Hosokawa T Hosono M Hanada K Aoike A Kawai K Takeda T Immune responses in newly developed short-lived SAM mice. Selectively impaired T-helper cell activity in in vitro antibody response Immunology 1987 62 3 425 429 2959613 PMC1454115

[B79] Hosokawa T Hosono M Higuchi K Aoike A Kawai K Takeda T Immune responses in newly developed short-lived SAM mice. I. Age-associated early decline in immune activities of cultured spleen cells Immunology 1987 62 3 419 423 3499380 PMC1454131

[B80] Kurozumi M Matsushita T Hosokawa M Takeda T Age-related changes in lung structure and function in the senescence-accelerated mouse (SAM): SAM-P/1 as a new murine model of senile hyperinflation of lung Am J Respir Crit Care Med 1994 149 3 Pt 1 776 782 8118649 10.1164/ajrccm.149.3.8118649

[B81] Ogawa H Renal lesions of the senescence accelerated mouse (SAM), with special emphasis on senility Nihon Jinzo Gakkai Shi 1988 30 9 1063 1065 3216547

[B82] Takeshita S Hosokawa M Irino M Higuchi K Shimizu K Yasuhira K Takeda T Spontaneous age-associated amyloidosis in senescence-accelerated mouse (SAM) Mech Ageing Dev 1982 20 1 13 23 10.1016/0047-6374(82)90070-7 7176700

[B83] Stanton H Rogerson FM East CJ Golub SB Lawlor KE Meeker CT Little CB Last K Farmer PJ Campbell IK Fourie AM Fosang AJ ADAMTS5 is the major aggrecanase in mouse cartilage in vivo and in vitro Nature 2005 434 7033 648 652 10.1038/nature03417 15800625

[B84] Malfait AM Liu RQ Ijiri K Komiya S Tortorella MD Inhibition of ADAM-TS4 and ADAM-TS5 prevents aggrecan degradation in osteoarthritic cartilage J Biol Chem 2002 277 25 22201 22208 10.1074/jbc.M200431200 11956193

[B85] Li J Anemaet W Diaz MA Buchanan S Tortorella M Malfait AM Mikecz K Sandy JD Plaas A Knockout of ADAMTS5 does not eliminate cartilage aggrecanase activity but abrogates joint fibrosis and promotes cartilage aggrecan deposition in murine osteoarthritis models J Orthop Res 2011 29 4 516 522 10.1002/jor.21215 21337391

[B86] Chen WH Hosokawa M Tsuboyama T Ono T Iizuka T Takeda T Age-related changes in the temporomandibular joint of the senescence accelerated mouse. SAM-P/3 as a new murine model of degenerative joint disease Am J Pathol 1989 135 2 379 385 2782378 PMC1879928

[B87] Kozyrev SV Abelson AK Wojcik J Zaghlool A Linga Reddy MV Sanchez E Gunnarsson I Svenungsson E Sturfelt G Jonsen A Truedsson L Pons-Estel BA Witte T D'Alfonso S Barizzone N Danieli MG Gutierrez C Suarez A Junker P Laustrup H Gonzalez-Escribano MF Martin J Abderrahim H Alarcon-Riquelme ME Functional variants in the B-cell gene BANK1 are associated with systemic lupus erythematosus Nat Genet 2008 40 2 211 216 10.1038/ng.79 18204447

[B88] Orozco G Abelson AK Gonzalez-Gay MA Balsa A Pascual-Salcedo D Garcia A Fernandez-Gutierrez B Petersson I Pons-Estel B Eimon A Paira S Scherbarth HR Alarcon-Riquelme M Martin J Study of functional variants of the BANK1 gene in rheumatoid arthritis Arthritis Rheum 2009 60 2 372 379 10.1002/art.24244 19180476

[B89] Shimada A Ohta A Akiguchi I Takeda T Inbred SAM-P/10 as a mouse model of spontaneous, inherited brain atrophy J Neuropathol Exp Neurol 1992 51 4 440 450 10.1097/00005072-199207000-00006 1619443

[B90] Shimada A Ohta A Akiguchi I Takeda T Age-related deterioration in conditional avoidance task in the SAM-P/10 mouse, an animal model of spontaneous brain atrophy Brain Res 1993 608 2 266 272 10.1016/0006-8993(93)91467-7 8495361

[B91] Chang MS Lowe DG Lewis M Hellmiss R Chen E Goeddel DV Differential activation by atrial and brain natriuretic peptides of two different receptor guanylate cyclases Nature 1989 341 6237 68 72 10.1038/341068a0 2570358

[B92] de Bold AJ Atrial natriuretic factor: a hormone produced by the heart Science 1985 230 4727 767 770 10.1126/science.2932797 2932797

[B93] Sudoh T Kangawa K Minamino N Matsuo H A new natriuretic peptide in porcine brain Nature 1988 332 6159 78 81 10.1038/332078a0 2964562

[B94] Simonnet G Allard M Legendre P Gabrion J Vincent JD Characteristics and specific localization of receptors for atrial natriuretic peptides at non-neuronal cells in cultured mouse spinal cord cells Neuroscience 1989 29 1 189 199 10.1016/0306-4522(89)90342-4 2540450

[B95] Teoh R Kum W Cockram CS Young JD Nicholls MG Mouse astrocytes possess specific ANP receptors which are linked to cGMP production Clin Exp Pharmacol Physiol 1989 16 4 323 327 10.1111/j.1440-1681.1989.tb01566.x 2545396

[B96] Hasegawa-Ishii S Takei S Inaba M Umegaki H Chiba Y Furukawa A Kawamura N Hosokawa M Shimada A Defects in cytokine-mediated neuroprotective glial responses to excitotoxic hippocampal injury in senescence-accelerated mouse Brain Behav Immun 2011 25 1 83 100 10.1016/j.bbi.2010.08.006 20804842

[B97] Zhu BH Ueno M Matsushita T Fujisawa H Seriu N Nishikawa T Nishimura Y Hosokawa M Effects of aging and blood pressure on the structure of the thoracic aorta in SAM mice: a model of age-associated degenerative vascular changes Exp Gerontol 2001 36 1 111 124 10.1016/S0531-5565(00)00179-0 11162916

[B98] Beyer EC Paul DL Goodenough DA Connexin43: a protein from rat heart homologous to a gap junction protein from liver J Cell Biol 1987 105 6 Pt1 2621 2629 2826492 10.1083/jcb.105.6.2621 PMC2114703

[B99] Beyer EC Kistler J Paul DL Goodenough DA Antisera directed against connexin43 peptides react with a 43-kD protein localized to gap junctions in myocardium and other tissues J Cell Biol 1989 108 2 595 605 10.1083/jcb.108.2.595 2537319 PMC2115444

[B100] Britz-Cunningham SH Shah MM Zuppan CW Fletcher WH Mutations of the Connexin43 gap-junction gene in patients with heart malformations and defects of laterality N Engl J Med 1995 332 20 1323 1329 10.1056/NEJM199505183322002 7715640

[B101] Dasgupta C Martinez AM Zuppan CW Shah MM Bailey LL Fletcher WH Identification of connexin43 (alpha1) gap junction gene mutations in patients with hypoplastic left heart syndrome by denaturing gradient gel electrophoresis (DGGE) Mutat Res 2001 479 1–2 173 186 11470490 10.1016/s0027-5107(01)00160-9

[B102] Blackburn JP Connat JL Severs NJ Green CR Connexin43 gap junction levels during development of the thoracic aorta are temporally correlated with elastic laminae deposition and increased blood pressure Cell Biol Int 1997 21 2 87 97 10.1006/cbir.1996.0122 9080656

[B103] Little TL Beyer EC Duling BR Connexin 43 and connexin 40 gap junctional proteins are present in arteriolar smooth muscle and endothelium in vivo Am J Physiol 1995 268 2 Pt 2 H729 H739 7864199 10.1152/ajpheart.1995.268.2.H729

[B104] Liao Y Regan CP Manabe I Owens GK Day KH Damon DN Duling BR Smooth muscle-targeted knockout of connexin43 enhances neointimal formation in response to vascular injury Arterioscler Thromb Vasc Biol 2007 27 5 1037 1042 10.1161/ATVBAHA.106.137182 17332489

